# Is Nigeria winning the battle against malaria? Prevalence, risk factors and KAP assessment among Hausa communities in Kano State

**DOI:** 10.1186/s12936-016-1394-3

**Published:** 2016-07-08

**Authors:** Salwa Dawaki, Hesham M. Al-Mekhlafi, Init Ithoi, Jamaiah Ibrahim, Wahib M. Atroosh, Awatif M. Abdulsalam, Hany Sady, Fatin Nur Elyana, Ado U. Adamu, Saadatu I. Yelwa, Abdulhamid Ahmed, Mona A. Al-Areeqi, Lahvanya R. Subramaniam, Nabil A. Nasr, Yee-Ling Lau

**Affiliations:** Department of Parasitology, Faculty of Medicine, University of Malaya, 50603 Kuala Lumpur, Malaysia; School of Health Technology, Club Road, Nassarawa, Kano, Kano State Nigeria; Endemic and Tropical Diseases Unit, Medical Research Center, Jazan University, Jazan, Kingdom of Saudi Arabia; Department of Parasitology, Faculty of Medicine and Health Sciences, Sana’a University, Sana’a, Yemen; North West Zonal Tuberculosis Reference Laboratory, Aminu Kano Teaching Hospital, Kano, Kano State Nigeria; Rabi’u Musa Kwankwaso College of Advanced and Remedial Studies, Tudun Wada, Kano State Nigeria; Department of Biology, Faculty of Natural and Applied Sciences, Umaru Musa Yar’adua University, Katsina, Katsina State Nigeria

**Keywords:** Malaria, Epidemiology, Prevalence, Knowledge, Attitude, Practice, Nigeria

## Abstract

**Background:**

Malaria is one of the most severe global public health problems worldwide, particularly in Africa, where Nigeria has the greatest number of malaria cases. This community-based study was designed to investigate the prevalence and risk factors of malaria and to evaluate the knowledge, attitudes, and practices (KAP) regarding malaria among rural Hausa communities in Kano State, Nigeria.

**Methods:**

A cross-sectional community-based study was conducted on 551 participants from five local government areas in Kano State. Blood samples were collected and examined for the presence of *Plasmodium* species by rapid diagnostic test (RDT), Giemsa-stained thin and thick blood films, and PCR. Moreover, demographic, socioeconomic, and environmental information as well as KAP data were collected using a pre-tested questionnaire.

**Results:**

A total of 334 (60.6 %) participants were found positive for *Plasmodium falciparum*. The prevalence differed significantly by age group (*p* < 0.01), but not by gender or location. A multivariate analysis showed that malaria was associated significantly with being aged 12 years or older, having a low household family income, not using insecticide treated nets (ITNs), and having no toilets in the house. Overall, 95.6 % of the respondents had prior knowledge about malaria, and 79.7, 87.6 and 95.7 % of them knew about the transmission, symptoms, and prevention of malaria, respectively. The majority (93.4 %) of the respondents considered malaria a serious disease. Although 79.5 % of the respondents had at least one ITN in their household, utilization rate of ITNs was 49.5 %. Significant associations between the respondents’ knowledge concerning malaria and their age, gender, education, and household monthly income were reported.

**Conclusions:**

Malaria is still highly prevalent among rural Hausa communities in Nigeria. Despite high levels of knowledge and attitudes in the study area, significant gaps persist in appropriate preventive practices, particularly the use of ITNs. Innovative and Integrated control measures to reduce the burden of malaria should be identified and implemented in these communities. Community mobilization and health education regarding the importance of using ITNs to prevent malaria and save lives should be considered.

## Background

Malaria continues to be a major public health problem in 97 countries and territories in the tropics and subtropics. Globally, approximately 214 million cases of malaria occur annually and 3.2 billion people are at risk of infection [1]. Approximately 438,000 deaths were attributed to malaria in 2015, particularly in sub-Saharan Africa, where an estimated 90 % of all malaria deaths occur [1]. As a critical target of the Millennium Development Goals, in 2005, the World Health Assembly established a goal of reducing malaria cases and deaths by 75 % between 2005 and 2015 [2]. Hence, over the past decade, there has been greatly renewed interest in research and innovations in diagnostic methods, drugs and vaccines, and the development of control measures to eradicate malaria [3]. As a result, between 2000 and 2013, the incidence rates of malaria fell by 30 % globally, and by 34 % in Africa [4].

Nigeria suffers the world’s greatest malaria burden, with approximately 51 million cases and 207,000 deaths reported annually (approximately 30 % of the total malaria burden in Africa), while 97 % of the total population (approximately 173 million) is at risk of infection [5]. Moreover, malaria accounts for 60 % of outpatient visits to hospitals and led to approximately 11 % maternal mortality and 30 % child mortality, especially among children less than 5 years [5, 6]. Malaria is caused by *Plasmodium falciparum*, and the mosquitoes *Anopheles gambiae*, *Anopheles funestus*, *Anopheles arabiensis*, and *Anopheles moucheti* are the major vectors that cause year-round transmission; artemether-lumefantrine (AL) or artesunate + amodiaquine (AS + AQ) is the treatment regime adopted in 2004 [1, 7]. This devastating disease affects the country’s economic productivity, resulting in an estimated monetary loss of approximately 132 billion Naira (~700 million USD), in treatment costs, prevention, and other indirect costs [8, 9].

Since 2008, the National Malaria Control Programme (NMCP) in Nigeria has adopted a specific plan, the goal of which is to reduce 50 % of the malaria burden by 2013 by achieving at least 80 % coverage of long-lasting impregnated mosquito nets (LLINs), together with other measures, such as 20 % of houses in targeted areas receiving indoor residual spraying (IRS), and treatment with two doses of intermittent preventative therapy (IPT) for 100 % of pregnant women who visit antenatal care clinics [10–12]. Because of these measures, the percentage of households with at least one LLIN increased to over 70 % by 2010, compared to only 5 % in 2008 [13–15]. Although previous studies have documented a high prevalence of malaria throughout Nigeria [16–21], there remains a paucity of research on people’s knowledge, attitudes, and practices (KAP) towards malaria in the majority of the federation, particularly in Northern Nigeria, including Kano State. This information is imperative in order to identify and implement effective control measures, and plan for the participation of the targeted communities in the control, which is one of the cardinal tools for the success and sustainability of disease control programmes [22–24]. Therefore, this study was designed to investigate the current prevalence and risk factors of malaria and to evaluate the people’s KAP regarding malaria in Kano State, North Central Nigeria.

## Methods

### Study design

A cross-sectional, community-based study was carried out between May and June 2013 among inhabitants aged 1–90 years in the five rural communities. Primary healthcare personnel and community leaders were involved in the study, and discussions were held with the households’ heads at the community leaders’ hall to explain the rationale and importance of the study.

### Study area

Kano State is located in the North Central part of the country between longitude 8.500°E and latitude 11.500°N; it occupies a total surface area of 20,131 km^2^ (77,773 m^2^) and has a total population of approximately 11 million. Kano State is a commercial and agricultural region known for the production of groundnuts and cotton. It is also the second largest industrial center in Nigeria, with textile, tanning, footwear, cosmetics, plastic, and other industries. The state consists primarily of Sudan savanna type vegetation, with an annual mean rainfall of 800-900 mm, a temperature that ranges between 25–40 °C (mean approximately 26 °C), and a relative humidity of 47.43 %. The climate of the study area is a tropical dry-and-wet season type typical of West African savannah. The wet season lasts from May to October, while the dry season extends from November to April [25].

The ecology of Nigeria varies from mangrove swamps and tropical rain forest belts in the coastal areas to open savannah woodland on the low plateau—which extends through much of the central part of the country—to semi-arid plains and Sahel grassland in the north and the eastern highlands. The seasonality, intensity, and duration of malaria transmission vary according to the different ecological strata [26]. The duration of malaria transmission increases from approximately 3 months in the north to year-round in the south. Moreover, the highest anthropophagic index and sporozoite positivity for the major malaria vectors was reported in the savanna forest region [27].

In collaboration with primary healthcare personnel and traditional rulers in each local government area, five districts were selected randomly from the available district list. The districts included were Kura (8.429°E, 11.774°N), Bebeji (8.400°E, 12.366°N), Gwarzo (7.932°E, 11.915°N), Shanono (7.983°E, 12.049°N), and Minjibir (8.530°E, 12.226°N).

### Study population

Residents of Kano State are predominantly farmers and merchants, and the districts selected have a homogenous nature with respect to sociocultural and daily economic activities. In the selected areas, primary healthcare staff and leaders of the communities were informed about the objectives and procedures of the study in order to obtain their permission to conduct the study and established a station. Then, the community leaders asked all residents to gather at the station (community leader’s hall) where they received explanation about the objectives of the study and their involvement. All residents who agreed voluntarily to participate were included in this study (universal sampling). A total of 609 individuals volunteered to participate in this study; however, 551 (90.5 %) individuals, aged 1–90 years, met the inclusion criteria (written signed consent, completed questionnaire, and blood samples for examination).

### Ethical considerations

Ethical approval for the study protocol was obtained from the Medical Ethics Committee of the University of Malaya Medical Centre, Kuala Lumpur as well as from Kano State’s Ministry of Health, Kano State Hospitals Management Board, the respective local government authorities, and the district heads of the communities. When seeking consent from the volunteers in each village, the objectives and procedures of the study were explained clearly to them in the local language, Hausa. Participants were also informed that they could withdraw from the study at any time without consequences. Thus, written and signed or thumb-printed informed consents were obtained from all adult participants and guardians/parents on behalf of their children before starting the survey; the ethics committees approved these procedures as well. All malaria-positive individuals were treated with the standard medication according to national malaria drug policy.

### Questionnaire survey

A pre-tested questionnaire was administered to the participants in order to collect information on their demographic, socioeconomic, and environmental factors, and the health status, as well as their KAP towards malaria. Information was collected from the respondents via face-to-face interviews conducted by trained research assistants. Both assistants were aware of the purpose of the study and the way in which to administer the questionnaire.

The survey gathered information offered both spontaneously and in response to specific questions that addressed their knowledge about malaria. Questions about knowledge were open-ended to avoid guessing about the answers to multiple-choice questions, which might give a false impression concerning the participants’ knowledge. However, questions pertaining to practices were multiple-choice to assess the frequency with which participants’ performed the various activities and actions. At the end of each interview, the interviewers probed for further knowledge related to malaria that the respondents did not mention spontaneously. However, this was done only for selected variables, specifically their source(s) of information about the disease, history, and reasons for previous visits to clinics or hospitals, as well as the availability, condition, and use of ITNs. During the survey, direct observations were made concerning the use of ITNs, as well as housing conditions and cleanliness (including the availability of functioning toilets, indoor plumbing, water containers, and cisterns), and status of windows.

### Blood sampling and examination

Approximately 2–3 ml of venous blood was drawn from each participant into an EDTA tube. Thick and thin blood films were prepared and a drop of the blood was used with an RDT kit, the “CareStart Malaria HRP2 from Access Bio, Inc.” Moreover, 2–3 drops were collected on 3 MM Whatman^®^ filter paper (Whatman International Ltd., Maidstone, England) and kept in individual sealed plastic bags at room temperature until use. At Aminu Kano Teaching Hospital, Kano State, the blood films were stained with diluted Giemsa and then examined microscopically for the presence of malaria parasites; 200 fields under 1000× magnification were examined from the thick film before the slide was considered negative.

For positive slides, parasite species and stages were assessed and parasitaemia (parasite density) was determined by counting only the asexual stages against 300 white blood cells (WBC) and then multiplying by 25, assuming the mean total WBC count of individuals is 7500 cells per μl of blood [28]. The level of parasitaemia was recorded as low (<1000 parasites/μl of blood), moderate (1000–9999 parasites/μl of blood), and severe (≥10,000 parasites/μl of blood).

### Molecular identification and genotyping

All malaria microscopy-positive slides and a random selection of 20 % of the negative slides were re-examined by another senior specialist at the Department of Parasitology, Faculty of Medicine, University of Malaya, Kuala Lumpur. Afterwards, all those samples were confirmed by PCR. Genomic DNA was extracted using the Qiagen blood and tissue kit (QIAGEN, DNeasy^®^ Blood & Tissue Kit, Cat. no. 69506, Germany); the filter paper blood spot was cut using an ethanol flame–sterilized hole-punch, put into a new 1.5 ml microcentrifuge tube, and then extraction was conducted according to the manufacturer’s instructions. DNA was eluted using 50 µl AE (10 mM Tris–Cl; 0.5 mM EDTA; pH 9.0) elution buffer (included in the kit) and maintained at −20 °C until PCR analysis. *Plasmodium species* were identified by 18s rRNA-based nested PCR using genus- and species-specific nucleotides primer sets, as described previously [29].

During the survey, all RDT-positive individuals were treated for malaria according to the national malaria drug policy. Afterward, study participants were considered to have falciparum malaria if the positive RDT results were confirmed by blood smears microscopy or PCR. Participants with positive PCR results but negative blood smears microscopy were considered to harbour sub-microscopic parasitaemias and were recorded as positive. Moreover, those who were positive by RDT but negative by microscopy and PCR were considered negative as RDT may give false positive results in individuals with a recent history of malaria infection or due to other infections such as trypanosomiasis, toxoplasmosis, hepatitis C, schistosomiasis, dengue and leishmaniasis [30]. Overall, none of the RDT-negative cases was identified as positive by microscopy or PCR and this is consistent with a recent evaluation for RDT conducted in Nigeria [31].

### Statistical analysis

Data were double entered by two different researchers into spreadsheets of SPSS, v. 20 (IBM Corporation, NY, USA). Then, a third researcher crosschecked the two datasets for accuracy and created a single dataset for analysis. The demographic, socioeconomic, environmental, and behavioural characteristics of the respondents, as well as the KAP variables were treated as categorical variables and presented as frequencies and percentages.

Pearson’s Chi squared test and Fisher’s exact test were used to test the associations between malaria prevalence and KAP items as the dependent variables, with the demographic (age, gender, and family size) and socioeconomic factors (educational and employment status, household monthly income, living near water sources, presence of functioning toilet in the house, housing conditions, using ITNs and insecticide, history of infection, and presence of domestic animals in the households) as the explanatory variables. All variables were coded as binary dummy variables. For example, malaria (positive = 1, negative = 0); gender (males = 1, females = 0), and so on. Odds ratios (OR) and 95 % confidence intervals (CI) were also computed for the explanatory variables. Moreover, participants were categorized into three age groups (<12, 12–17 and 18 years and older); however, they were re-categorized into two groups (children <18 years and adults 18 years and older) for analysis of the KAP variables. ORs at 95 % CIs were also computed. Multivariable logistic regression was conducted to identify the risk factors associated significantly with infection. To retain all possible significant associations, all variables that showed an association with *p* ≤ 0.25 were used in the multiple logistic regression model, as suggested by Bendel and Afifi [32]. A *p* < 0.05 was considered statistically significant.

## Results

A total of 551 individuals participated in the study; 340 (61.7 %) were male, 211 (38.3 %) were female, and 64.1 % were adults aged 18 years and older. Table 1 shows the general demographic, socioeconomic, and environmental characteristics of the study participants. Among these, 23.0 % were inhabitants of Kura, 21.6 % of Bebeji, 17.6 % of Gwarzo, 18.0 % Shanono, and 19.9 % of Minjibir rural areas. Generally, the state government’s minimum wage is N18,000 (112.5 USD) per head; however, only 41.9 % of the respondents had a household monthly income of N32,000 (200 USD). In these communities, the houses were predominantly of traditional construction, with mud walls, but zinc roofing. The water supply was unstable; nevertheless, 64.6 % had drinking water, and 63.2 % used safe water for other domestic chores as well. The majority of the participants had LLIN distributed freely by the NMCP.

**Table 1:** General characteristics of the participants (n = 551)

Variables	N	%
Gender		
Male	340	61.7
Female	211	38.3
Age groups (years)		
<12	63	11.4
12–17	135	24.5
≥18	353	64.1
Address		
Kura	127	23.0
Bebeji	119	21.6
Gwarzo	97	17.6
Shanono	99	18.0
Minjibir	109	19.9
Household monthly income		
<NGN 32,000	231	41.9
≥NGN 32,000	320	58.1
Type of house		
Mud	430	78.0
Concrete	121	22.0
Water source		
Safe drinking water	356	64.6
Safe domestic water	348	63.2
*Presence of domestic animals*	228	41.4
Insecticide treated nets (ITNs)		
Having ITNs	438	79.5
Do not have ITNs	113	20.5
Source of ITNs		
Government	363	83.1
Personal	75	16.9
History of malaria infection		
Recent (less than 6 months)	183	33.2
Long ago	368	66.8

*NGN* Nigerian Naira; (US$1 = NGN 165)

### Prevalence and distribution of malaria

The prevalence of malaria infection was 60.6 % (334/551: Table 2). Overall, 15 RDT-positive cases were found negative by microscopy; however, five of them were confirmed positive by PCR. All infections were of *P. falciparum* and the majority (70.1 %) constituted low parasitaemia (<1000 parasites/μl of blood). Moreover, 10 (3.0 %) and 90 (26.9 %) cases were severe (≥10,000 parasites/μl of blood) and moderate (1000–9999 parasites/μl of blood), respectively. The asexual *P. falciparum* parasitaemia from the samples collected ranged from 50–12,500 parasites/μl of blood, with a geometric mean of 1023 parasites/μl. Overall, the prevalence of malaria was similar between males and females (61.2 vs 59.7 %; Chi square (χ^2^) = 0.12, *p* > 0.05). Similarly, there was no significant difference in prevalence according to location (χ^2^ = 2.31, *p* > 0.05). However, the prevalence differed significantly (χ^2^ = 9.40; *p* < 0.01) among age groups, with those aged 12–17 years having the highest prevalence (66.7 %). Among those aged less than 12 years, the overall prevalence was 42.9 % with those aged below 5 years having a prevalence of 37.5 %.

**Table 2:** Prevalence and distribution of malaria among the participants according to age, gender and location (n = 551)

Variables	No. examined	Malaria-positive	Recent history of malaria	Using ITNs
	N	n (%)	n (%)	n (%)
Age groups (years)				
<12	63	27 (42.9)	39 (61.9)	38 (60.3)
12–17	135	90 (66.7)	99 (73.3)	50 (37.0)
≥18	353	217 (61.5)	230 (65.2)	185 (52.4)
χ^2^		10.502	3.709	12.531
*p*		0.005	0.157	0.002
Gender				
Male	340	208 (61.2)	109 (32.1)	165 (48.5)
Female	211	126 (59.7)	74 (35.1)	108 (51.2)
χ^2^		0.116	0.533	0.367
*p*		0.733	0.466	0.545
Location				
Kura	127	74 (58.3)	45 (35.4)	70 (55.1)
Bebeji	119	68 (57.1)	51 (42.9)	54 (45.4)
Gwarzo	97	62 (63.9)	36 (37.1)	25 (25.8)
Shanono	99	59 (59.6)	24 (24.2)	62 (62.6)
Minjibir	109	71 (65.1)	27 (24.3)	62 (56.9)
χ^2^		2.314	13.031	33.455
*p*		0.678	0.011	<0.001
Total	551	334 (60.6)	183 (33.2)	273 (49.5)

With regard to history of infection, approximately one-third (33.2 %) of the participants had experienced a recent malaria episode. A significant association was found between this history of infection and location (χ^2^ = 13.03, *p* < 0.05); Bebeji had the highest percentage (42.9 %) and Shanono the lowest (24.2 %). Approximately half of the respondents (49.5 %; 273/551) reported that they used ITNs, and there was a significant difference in use between age groups (*p* < 0.01) and location (*p* < 0.001), with participants aged 12–17 years (37.0 %), and those from Gwarzo (25.8 %) having the lowest percentage of use; there was no significant difference between males and females (*p* > 0.5).

### Risk factors of malaria

Table 3 shows the results of the univariate analysis of the association between malaria and the potential explanatory variables. The results showed that age group, low family monthly income, and use of ITNs were associated significantly with malaria prevalence. A significantly higher prevalence was reported among participants aged 12 years and above (*p* < 0.01), those from families with low monthly income (<NGN 32,000) compared to their counterparts (67.1 vs 55.9 %; *p* < 0.05), and those who did not use ITNs compared to those who did (66.5 vs 54.6 %; *p* < 0.01).

**Table 3:** Univariate and multivariate analyses of potential risk factors associated with malaria among the Nigerian participants (n = 551)

Variables	Malaria			*P*
	No. examined	% infected	OR (95 % CI)	
Age groups				
≥18	353	61.5	2.13 (1.24, 3.66)	0.006*^, †^
12–17	135	66.7	2.67 (1.44, 4.93)	0.002*^, †^
<12 years	63	42.9	1	
Gender				
Male	340	61.2	1.06 (0.75, 1.51)	0.733
Female	211	59.7	1	
Educational status				
Not educated	88	54.5	0.76 (0.46, 1.27)	0.289
Primary education	272	62.1	1.04 (0.71, 1.52)	0.849
Secondary/tertiary education	191	61.3	1	
Occupation				
Working	270	62.6	1.18 (0.84, 1.66)	0.352
Not working	281	58.7	1	
Household monthly income				
<NGN 32,000 (low)	231	67.1	1.61 (1.13, 2.29)	0.008*^, †^
≥NGN 32,000	320	55.9	1	
Family size				
>10 members	284	60.9	1.01 (0.85, 1.21)	0.882
≤10 members	267	60.3	1	
Type of houses				
Mud	430	60.5	0.97 (0.64, 1.47)	0.891
Concrete	121	61.2	1	
Type of toilet				
Pit (ground dug)	481	61.7	1.44 (0.87, 2.38)	0.155^†^
Pour flush system	70	52.9	1	
Having ITNs				
No	113	61.1	1.01 (0.92, 1.10)	0.914
Yes	438	60.5	1	
Using ITNs				
No	278	66.5	1.66 (1.17, 2.34)	0.004*^, †^
Yes	273	54.6	1	
Using insecticide				
No	348	61.5	1.07 (0.85, 1.33)	0.581
Yes	203	59.1	1	
Have domestic animals				
Yes	228	57.9	0.82 (0.58, 1.16)	0.272
No	323	62.5	1	
Living near water sources (stream, dam, lake, pond, etc.)				
Yes (<250 m)	380	60.0	0.92 (0.64, 1.33)	0.659
No	171	62.0	1	
Recent history of infection				
Yes	183	59.0	0.97 (0.86, 1.09)	0.588
No	368	61.4	1	

*NGN* Nigerian Naira; (US$1 = NGN 165); *OR* odds ratio; *CI* confidence interval

* Significant association (*p* < 0.05)

^†^ Confirmed as significant risk factors by multiple logistic regression analysis

The multiple logistic regression showed that participants aged 12 years and older were approximately twice as likely to have malaria compared to those younger than 12 years (adjusted OR = 2.23; 95 % CI = 1.19, 4.17). Similarly, low household monthly income (<NGN 32,000) was retained as a significant risk factor and was found to increase the odds of infection by 1.61 times (95 % CI = 1.14, 2.29). Moreover, it was found that not using ITNs increased participants’ odds for malaria by 1.57 times (95 % CI = 1.10, 2.24) when compared to those who used them. Interestingly, the type of toilet was considered in the regression model, based on the *p* < 0.25 [30], and was retained as the fourth significant risk factor of malaria (OR = 1.70; 95 % CI = 1.00, 2.88; *p* = 0.049).

### Knowledge, attitudes, and practices regarding malaria

Knowledge of the cause, transmission, symptoms, and prevention of malaria, as well as the perception of its seriousness, is presented in Table 4. Generally, the respondents were well informed about malaria; 483 (95.6 %) knew about malaria (excluding children less than 10 years; thus n = 505). Knowledge about malaria was obtained primarily through personal/relatives’ experiences (48.9 %) or the media (27.3 %). Approximately one-fifth of the respondents (20.3 %) did not know the cause of malaria, but the majority (77.8 %) ascribed it to mosquitoes. Approximately three-quarters (77.8 %) of the respondents mentioned fever and slightly less than half mentioned weakness (48.2 %) as symptoms of malaria. Moreover, 69.6 % of the respondents indicated that they avoided mosquitoes by using bed nets or insecticide, while only 4.3 % replied that they did not know how to prevent malaria.

**Table 4:** Knowledge, attitude and practices of respondents with regards to malaria in Kano State, Nigeria

Variables	n	%
Knowledge (n = 505)		
Know malaria	483	95.6
Source of information (n = 483)		
Media (TV, radio, newspaper)	132	27.3
Awareness campaign	56	11.6
Family and friends	236	48.9
Do not know	59	12.2
Causes (n = 483)		
Mosquito bites	364	75.3
Stagnant water	12	2.5
Poor personal hygiene	9	1.9
Do not know	98	20.3
Signs and symptoms (n = 483)		
Fever	376	77.8
Headache	50	10.4
Weakness	233	48.2
Vomiting	55	11.4
Abdominal pain	49	10.1
I do not know	60	12.4
Prevention (n = 483)		
Using bed nets/insecticide	336	69.6
Improved personal hygiene	103	21.3
Medication	34	7.0
Do not know	21	4.3
Attitude (n = 483)		
Serious disease	451	93.4
Not serious disease	11	2.3
Do not know	21	4.3
Practices (n = 505)		
Having ITNs	395	78.2
Using ITNs	244	48.3
Using insecticide	189	37.4
Houses with wood/zinc roofs	392	77.6
Houses with mud wall	284	56.2
Living near water sources (stream, dam, lake, pond, etc.)	348	68.9
Treatment seeking behaviour (n = 505)		
Hospitals/clinics	375	74.3
Self medication	97	19.2
Traditional/herbal medicine	14	2.8
Do nothing	19	3.8

With respect to attitudes, most subjects (93.4 %) regarded malaria as a serious infection. The distribution of ITNs was high in the study area, and 78.2 % of the respondents had at least one ITN in their homes. However, only 48.3 % of the 505 respondents used ITNs, and only one-third (37.4 %) of the respondents used insecticide. With regard to treatment seeking behaviour, a considerable number of the respondents (375, 74.3 %) mentioned that they went to hospitals or clinics when they had an episode of fever, while 19.2 % of the respondents self-medicated as a first-line of treatment for fever; 3.8 % of the respondents did not treat malaria infection.

Tables 5 and 6 show the association between the respondents’ knowledge and attitudes about malaria and their age, gender, educational status, and household monthly income. The results showed significantly higher levels of knowledge of malaria symptoms, particularly weakness, vomiting, and abdominal pain among adult respondents than children. Adult participants had significantly lower levels of knowledge about mosquito bites (72.6 vs 81.8 %) as a cause of malaria, as well as using bed nets/insecticide (65.9 vs 78.3 %) as a preventive measure. Overall, the adults had better attitudes about the seriousness of malaria compared to those younger than 18 (95.6 vs 88.1 %).

**Table 5:** Association of participants’ knowledge about malaria with their age and gender

Variables	Age (years)				Gender			
	<18	≥18	OR	95 % CI	Female	Male	OR	95 % CI
Heard about malaria	143 (94.1)	340 (96.3)	1.65	0.69, 3.94	184 (94.4)	299 (96.5)	1.63	0.69, 3.82
Causes								
Mosquito bites	117 (81.8)	247 (72.6)	0.59	0.36, 0.96*	117 (63.6)	247 (82.6)	2.72	1.78, 4.16*
Stagnant water	3 (2.1)	9 (2.6)	1.27	0.34, 4.76	4 (2.2)	8 (2.7)	1.24	0.37, 4.17
Poor personal hygiene	4 (2.8)	5 (1.5)	0.52	0.14, 1.96	3 (1.6)	6 (2.0)	1.24	0.31, 5.01
Do not know	19 (13.3)	79 (23.2)	1.98	1.15, 3.41*	60 (32.6)	38 (12.7)	0.30	0.20, 0.48*
Signs and symptoms								
Fever	114 (79.7)	262 (77.1)	0.85	0.53, 1.38	127 (69.0)	249 (83.3)	2.24	1.45, 3.46*
Headache	12 (8.4)	38 (11.2)	1.37	0.70, 2.71	14 (7.6)	36 (12.0)	1.66	0.87, 3.17
Weakness	49 (34.3)	184 (54.1)	2.26	1.51, 3.40*	91 (49.5)	142 (47.5)	0.92	0.64, 1.34
Vomiting	6 (4.2)	49 (14.4)	3.85	1.61, 0.19*	18 (9.8)	37 (12.4)	1.30	0.72, 2.36
Abdominal pain	7 (4.9)	42 (12.4)	2.74	1.20, 6.25	23 (12.5)	26 (8.7)	0.67	0.37, 1.21
Do not know	15 (10.5)	45 (13.2)	1.30	0.70, 2.42	35 (19.0)	25 (8.4)	0.39	0.23, 0.67*
Prevention								
Using bed nets/insecticide	112 (78.3)	224 (65.9)	0.53	0.34, 0.84*	97 (52.7)	239 (79.9)	3.57	2.38, 5.36*
Improving personal hygiene	31 (21.7)	72 (21.2)	0.97	0.60, 1.56	20 (10.9)	83 (27.8)	3.15	1.86, 5.35*
Medication	10 (7.0)	24 (7.1)	1.01	0.50, 2.17	3 (1.6)	31 (10.4)	6.98	2.10, 23.17*
Do not know	6 (4.2)	15 (4.4)	1.05	0.40, 2.77	12 (6.5)	9 (3.0)	0.45	0.18, 1.08

All values are number (%)

*OR* odds ratio; *CI* confidence interval

* Significant association (*p* < 0.05)

**Table 6:** Association of participants’ knowledge about malaria with their educational status and monthly income

Variables	Educational status				Household monthly income			
	Not educated	Educated	OR	95 % CI	<GN 32,000	≥NGN 32,000	OR	95 % CI
Heard about malaria	65 (91.5)	418 (96.3)	2.41	0.91, 6.39	206 (94.9)	277 (96.2)	1.35	0.57, 3.16
Causes								
Mosquito bites	33 (50.8)	341 (81.6)	4.30	2.49, 7.41*	171 (83.0)	203 (73.3)	0.56	0.36, 0.88*
Stagnant water	3 (4.6)	19 (4.5)	0.98	0.28, 3.42	10 (4.9)	12 (4.3)	0.89	0.38, 2.10
Poor personal hygiene	45 (10.8)	2 (3.1)	3.80	0.90, 16.06	22 (10.7)	25 (9.0)	0.83	0.45, 1.52
Do not know	27 (41.5)	74 (17.7)	0.31	0.17, 0.53*	32 (15.5)	69 (24.9)	1.81	1.13, 2.87*
Signs and symptoms								
Fever	43 (66.2)	333 (79.7)	2.01	1.14, 3.53*	161 (78.2)	215 (77.6)	0.97	0.63, 1.50
Headache	9 (13.8)	43 (10.3)	0.73	0.48, 1.33	17 (8.3)	38 (13.7)	1.86	0.89, 2.43
Weakness	32 (49.2)	201 (48.1)	0.96	0.57, 1.61	95 (46.1)	138 (49.8)	1.16	0.81, 1.67
Vomiting	6 (9.2)	98 (23.4)	3.01	1.26, 7.19*	32 (15.5)	72 (26.0)	1.91	1.20, 3.03*
Abdominal pain	5 (7.7)	22 (5.3)	0.74	0.62, 1.25	19 (9.2)	31 (11.2)	1.14	0.73, 1.21
Do not know	12 (18.5)	50 (12.0)	0.60	0.30, 1.20	28 (13.6)	34 (12.3)	0.89	0.52, 1.52
Prevention								
Using bed nets/insecticide	30 (46.2)	306 (73.2)	3.19	1.87, 5.44*	150 (72.8)	186 (67.1)	0.76	0.51, 1.13
Improving personal hygiene	8 (12.3)	95 (22.7)	2.10	0.97, 4.55	46 (22.3)	57 (20.6)	0.90	0.58, 1.40
Medication	2 (3.1)	32 (7.7)	2.61	0.61, 11.17	17 (8.3)	17 (6.1)	0.73	0.36, 1.46
Do not know	3 (4.6)	46 (11.0)	2.56	0.77, 8.47	24 (11.7)	25 (9.0)	0.75	0.42, 1.36

All values are number (%)

*OR* odds ratio; *CI* confidence interval

* Significant association (*p* < 0.05)

With respect to gender, male respondents showed significantly higher levels of knowledge about malaria, particularly mosquito bites (82.6 vs 63.6 %) as a cause; fever as a symptom (83.3 vs 69.0 %); using bed nets/insecticide (79.9 vs 52.7 %); improved personal hygiene (27.8 vs 10.9 %), and medication (10.4 vs 1.6 %) as preventive measures. Moreover, significantly higher levels of knowledge of the role of mosquitoes in malaria transmission (81.6 vs 50.8 %) as well as fever (79.7 vs 66.2 %) and vomiting (23.4 vs 9.2 %) as symptoms of malaria, and using bed nets/insecticide to prevent infection (73.2 vs 46.2 %) were reported among educated respondents compared to their non-educated counterparts (Table 6). In addition, respondents who had household monthly incomes of ≥NGN 32,000 had significantly lower levels of knowledge about the role of mosquitoes in disease transmission (73.3 vs 83.0 %) compared to those having low household monthly incomes.

## Discussion

This study reported high prevalence of malaria in Kano State as, where 60.6 % of the participants were found positive for *P.falciparum*. This is consistent with previous studies conducted in Kano and other Nigerian states. Moreover, recent malaria risk maps estimated that malaria prevalence in Nigeria varied from less than 20 % in certain areas to over 70 % in others [21]. This variation can be attributed to different climatic conditions, less rainfall, and surface water that serve as mosquito breeding sites. Previous studies from Kano State showed that 62.5 % (250/400) of the patients attending two hospitals in Kano Metropolis and 51.7 % (155/300) of the pregnant women attending antenatal clinic at a specialist hospital were malaria-positive [33, 34]. A similar high prevalence (61.3 %) was reported recently among pregnant women attending Aminu Kano Teaching Hospital [35]. In the same vein, over 22,000 deaths from malaria infection were reported in Kano State during 2015 [36]. These findings revealed that malaria continues to be a leading cause of morbidity and mortality in Kano, and suggested that innovative and effective interventions to control malaria should be identified and implemented in these communities.

Consistent with the present findings, previous studies among children in Kebbi, Awka, and Abuja States reported prevalence rates of 64.0 % (128/200), 59.6 % (118/198), and 58.0 % (233/400), respectively [20, 37, 38]. Similarly, previous studies reported *P. falciparum* prevalence rates of 62.4 and 57.1 % among pregnant women in Ogun and Niger Delta States, respectively [39, 40]. Nonetheless, higher prevalence rates were reported among pregnant women who visit primary healthcare facilities in Imo, Ogun, Gboko, Osun, and Benue States, where the prevalence rates were 80.1, 78.9, 76.9, 72 and 68.3 %, respectively [41–45]. Moreover, an extraordinary population-based prevalence rate (99.2 %) was reported among 2069 pregnant women in Enugu State, Southeastern Nigeria [46]. Similarly, a high prevalence rate (80 %) was reported among other populations in Southern Nigeria [16, 47].

In contrast, other previous studies reported lower prevalence rates: 39.2 % of 360 antenatal clients who visit primary healthcare facilities in Kano State [17], 29.4 % of 160 blood donors in Zaria State [18], 26 % of 400 pregnant women in Port-Harcourt, Rivers State [48], 35 % of 100 long distance truck drivers in Niger Delta State [49], 42.3 % in Otukpo, Benue state, 36.1 % (1060/2936) [50], and 36.6 % (1540/4209) in Abia and Plateau States, and 41.6 % (106/255) in pregnant women in a semi-urban community of Argungu, Kebbi State [51]. When compared to other sub-Saharan countries endemic for malaria, the prevalence reported in this study was higher than the 48.2, 47.8, 49.3 and 42.9 % reported in the Democratic Republic of the Congo [52], Mozambique [53], Burkina Faso [54], and Sierra Leone [55], respectively. Led by Nigeria, these countries are considered the top six among countries affected by malaria [56]. Similarly, a recent study revealed that 42.0 % of 2346 schoolchildren living in a high transmission area in Western Kenya were positive for *P. falciparum* [57].

Findings of the present study showed a similar prevalence rate among the male and female participants, which is consistent with previous studies in Nigeria, Kenya, and Mozambique, which suggests that the distribution of malaria risk is heterogeneous [19, 53, 58]. However, males were at higher risk of malaria infection due to exposure, and inherent and cultural determinants [59–62]. Coinciding with this, greater and repeated exposure to malaria among males may result in development of partial immunity that renders them at lower risk of clinical malaria compared to females [63], a situation reported in Western Kenya as well [57].

With respect to location, there were no significant differences in the prevalence of malaria among the districts studied; however, an obvious correspondence between a history of recent malaria and use of ITNs was observed, with the lowest percentage of history of recent malaria reported in Shanono (24.2 %), which also has the highest percentage of ITNs use (62.6 %). Due to the specific characteristics of populations that may facilitate human–mosquito contact, malaria risk can vary widely among districts, villages, and even households [64]. A recent study among children in Zambezia province, Mozambique showed that malaria prevalence varied among villages within the same province and ranged from 22.1 to 87.2 % [53].

The present study demonstrated that age, household monthly income, ITNs use and type of toilets were the significant risk factors associated with malaria among these participants. The present findings showed that prevalence was significantly higher among those aged 12–17 years (66.7 %) and ≥18 years (61.5 %) compared to those younger than 12 years (42.9 %). Interestingly, this difference could be explained by the reported age-specific use of ITNs; 60.3 % of those younger than 12 years compared to only 37.0 % of those aged 12–17 years used ITNs. Moreover, individuals aged 12 years and older are expected to have a higher exposure to mosquito bites because they engage in more outdoor activities compared to younger children, particularly at night. These findings are consistent with recent reports that rates of ITNs use were significantly higher among the youngest children, particularly those less than 5 years, and heads of the family, and this might be attributed to the inadequate ITNs per household [65]. Hence, these findings imply that the number of ITNs available to households should be increased to achieve universal coverage in these communities. Similarly, another study revealed that adolescent boys were least likely to use ITNs [15], while a recent study in Abia and Plateau States found that the prevalence of *Plasmodium* infection was associated significantly with age, with the highest prevalence among children 5–9 years [19]. In contrast, a recent study among Kenyan schoolchildren found that *P. falciparum* infection reduced with increasing age, and those aged 11–15 years had a 0.78 odds of infection compared to those aged 5–10 years [57].

The present study also showed that low household monthly income (<NGN 32,000) increased the odds of malaria among these children by approximately 1.5 times. This is consistent with previous reports from other African countries that showed malaria is more common among people of lower socioeconomic status who often live in poor housing conditions that increase their exposure to infection [57, 62, 66, 67]. Malaria and poverty are connected intimately, as malaria primarily affects low- and lower-middle income countries, where the poorest communities are affected most severely by malaria due to their poor socioeconomic and environmental status, and inadequate services for prevention, diagnosis, and treatment [5]. Hence, these endemic communities are trapped in a vicious cycle of poverty, underdevelopment, and disease [68]. Despite high IRS coverage and equitable ITN distribution, poverty at both the community and household levels was a significant risk factor for malaria in Northwestern Tanzania [69].

Use of ITNs is considered one of the most cost-effective interventions against malaria in endemic areas, and is associated with significant reductions in malaria morbidity and mortality, particularly among pregnant women and children less than 5 years [70]. The current study showed that failure to use ITNs was a significant risk factor for malaria among the population studied, which is consistent with previous studies in Nigeria and Ethiopia [51, 62].

Despite the high number of participants who had ITNs (79.5 %), only half of the participants stated that they used them, and were found to do so (49.5 %). This unsatisfactory compliance is consistent with previous studies in Nigeria [14, 19, 71–73] and other countries [74, 75]. A recent national study in Nigeria demonstrated a notable gap between ownership and use of ITNs and showed that, at the national level, only 28.7 % of the population had one in the household; this rate increased to 50.0 % in areas with a recent campaign (including Kano State) [72].

Interestingly, the present study showed that approximately one-third (30 %) of those who had ITNs and stated that they used them were found to misuse the nets; hence, they were considered non-users. The improper uses of the ITNs observed included employing bed covers, blankets, window curtains, and door curtains rather than actual ITNs. Moreover, some participants declared that they washed the ITNs before using them to remove the chemicals, which either have an unpleasant smell or induce allergies in some individuals. In addition, some of the ITNs were old and were not re-treated, while others were in poor condition. Similar misuse has been reported in different malaria-endemic African countries, including Nigeria, Uganda, Tanzania, Kenya, Zambia, Sierra Leone, and the Democratic Republic of Congo [76–81]. In addition to the misuses observed, a previous report indicated that some individuals who receive free ITNs frequently sell them on the open market, while many communities refuse to use them because of a misconception that they are purely a Western intervention that has been forced on African communities, with little regard for local norms or cultures [78]. Moreover, people do not use the ITNs because of a perception that malaria or mosquitoes are not a serious problem [76].

The present study also found that the type of toilet in the house was a significant risk factor for malaria in the communities studied, as those living in houses with pit or ground dug latrines were 1.7 times more likely to be infected with malaria than were those who have toilets in their houses with a pour/flush system. The lack of availability of in-home toilets has been reported to be a significant predictor of malaria in Ethiopia and Yemen [82, 83].

With respect to respondents’ KAP, findings the current study indicated that general awareness of malaria was high among Hausa communities in Kano State and almost all of them (95.6 %) had heard about malaria. This is expected, as malaria is considered the primary health problem in these communities [84–86]. Most of the respondents in the present study were well acquainted with malaria symptoms, and 77.8 % of them recognized fever as a detrimental consequence of malaria. Moreover, the present findings showed that the majority of the respondents had received information about malaria prevention, and using bed nets and/or insecticide, improving personal hygiene, and taking medication were the three main preventive measures they cited. This agrees with previous studies in other parts of Nigeria [87, 88], and other malaria-endemic countries [84, 86, 89]. However, a previous study among pregnant women from Ogun State in Southwest Nigeria showed that knowledge of malaria transmission and prevention was generally poor, in that only 36.3 % of the women associated malaria infection with mosquito bites, and none mentioned using ITNs as a preventive measure [39]. Similarly, a previous study among rural farming communities in Oyo State, Southwestern Nigeria, reported that only 12.4 % respondents were aware of the role of mosquito bites in transmitting the disease and less than half (46.7 %) were able to state at least one symptom of malaria [90]. Recent studies from Southwest Nigeria also revealed that knowledge of malaria remains low among caregivers of children less than five, pregnant women and mothers [80, 91].

The present study showed that the majority of the respondents (93.4 %) indicated that malaria is a serious disease, which is consistent with previous studies in Nigeria and other countries [84, 85, 88]. Moreover, this study showed that only a very small percentage (2.3 %) believed that malaria is not harmful. They claimed, “Malaria is not so serious because everyone has it, and cannot be cured.” Some said, “It sometimes causes fever, when one takes medication the fever goes away but the malaria does not, we are born with it.” Others stated, “Malaria cannot be cured, it always comes back,” and “Malaria is in our blood, it is always there, just avoid going down with fever by avoiding stress and always eat good food.” A few were indifferent, and believed that mosquito bites are simply a nuisance; it is good if they can be avoided, but otherwise they have no consequences. However, findings of the current study contradicted those of a recent study in Osun State, Southwest Nigeria, which demonstrated that the majority of the mothers believed that malaria is a simple disease for which they can comfortably apply home remedies [91]. Another hospital-based study about awareness of ITN use in Abeokuta State, Southwest Nigeria reported low awareness among the pregnant women interviewed [92].

Notwithstanding the high prevalence of attitudes that the disease is serious, approximately 20 % of the respondents tended to begin treatment at home and sought help from professionals only when it failed. Home treatment usually involved self-medication or unqualified prescription by family members, friends, and unauthorized shops. This behaviour did not different with malaria fever, wherein people began treatment with analgesics and then anti-malarial drugs if symptoms persisted. The same behaviour has been reported elsewhere in Nigeria [85, 93–95], and from other parts of Africa [96, 97]. Intriguingly, a previous study in Oyo state, Southwest Nigeria, showed that approximately 90 % of suspected malaria cases were self-treated first at home with traditional herbs or drugs purchased from medicine stores [85]. Moreover, a significant difference in treatment-seeking behaviour was reported between rural and urban mothers in Southeast Nigeria, in that two-thirds of urban mothers preferred private/government health facilities, while two-thirds of their rural counterparts preferred self-treatment with drugs bought over-the-counter from patent medicine vendors [94].

This study found that age, gender, educational level, and household monthly income had significant influences on having adequate knowledge about malaria. Younger respondents showed a greater level of knowledge about malaria transmission and prevention than did adults (aged 18 and older), which could be attributed to schools or media, especially television. Similarly, males had substantially higher levels of knowledge about malaria transmission, symptoms, and prevention, which could be because of their higher exposure, as well as certain behavioural and cultural factors. In Nigeria, the prevalence of malaria is highest among pregnant women compared to other groups [41, 42, 46]. Therefore, a health education programme about the disease that targets those women will result in a significant reduction in malaria in the country.

Similar high levels of knowledge about malaria transmission, and prevention were also reported among those having household monthly incomes below NGN 32,000 compared to their counterparts, which is inconsistent with the higher prevalence reported among those respondents. This suggests that the higher prevalence rate among this group is more related to the practices but not the knowledge. This finding also is consistent with previous studies in Nigeria [80, 90, 91]. Obviously, poverty hinders the efforts of malaria control programmes in the study area and other parts of Nigeria, which is worth noting, in that it can help policymakers target these specific populations and implement control measures. Previous studies in Nigeria have shown that the level of education was a strong predictor of positive malaria-related KAP [73, 85, 98]. Similarly, the choice of going to hospital on time depends on the level of education, as non-educated respondents are more likely to patronize unauthorized practitioners or buy medication from street vendors. This practice, combined with the poor quality of anti-malarial drugs in Africa, has led to the emergence and wide distribution of chloroquine-resistant malaria among the population [95, 99]. Therefore, policymakers should recognize that proper training of medical and paramedical personnel in malaria treatment and management must include those “shop operators,” as they are associated most closely with the population, and attitudes about the efficacy of treatment with their remedies cannot be altered immediately.

## Conclusions

This study revealed that Nigeria’s battle against malaria has a long way to go. A high prevalence of *falciparum* malaria was reported among rural populations in Kano State. Hence, there is an urgent need to identify innovative and integrated control measures to reduce malaria prevalence significantly in these communities. Age, household monthly income, use of ITNs, and availability of in-house toilets were the significant predictors of malaria in this population. The study demonstrated that respondents had good levels of knowledge and attitudes regarding malaria transmission, symptoms, and prevention; however, these did not translate into improved preventive practices. The present study found that, despite the high level of ITNs ownership, their use still fell short of the national target. Hence, additional strategies, including community mobilization and more focused health education on the importance of using ITNs to control malaria and save lives, are required to increase use and improve current levels of ownership. That said, the number of ITNs distributed to households should be increased to ensure that populations living in malaria-risk areas have enough ITNs for all households members. Moreover, long-term interventions, such as providing job opportunities and improving the level of education to reduce poverty and improve the quality of life of these vulnerable communities also should be considered in order to achieve a breakthrough in the fight against malaria in Nigeria.

## Abbreviations

KAP: knowledge, attitudes and practices
ITNs: insecticide-treated nets
WHO: World Health Organization
NMCP: National Malaria Control Programme
RDT: rapid diagnostic test
NGN: Nigerian Naira
